# Toxicity Rank Order (TRO) As a New Approach for Toxicity Prediction by QSAR Models

**DOI:** 10.3390/ijerph20010701

**Published:** 2022-12-30

**Authors:** Yuting Chen, Yuying Dong, Le Li, Jian Jiao, Sitong Liu, Xuejun Zou

**Affiliations:** College of Environment and Resource, Dalian Minzu University, Dalian 116600, China

**Keywords:** toxicity rank order (TRO), Quantitative Structure–Activity Relationship (QSAR), toxicity mechanisms, accuracy, validity

## Abstract

Quantitative Structure–Activity Relationship (QSAR) models are commonly used for risk assessment of emerging contaminants. The objective of this study was to use a toxicity rank order (TRO) as an integrating parameter to improve the toxicity prediction by QSAR models. TRO for each contaminant was calculated from collected toxicity data including acute toxicity concentration and no observed effect concentration. TRO values associated with toxicity mechanisms were used to classify pollutants into three modes of action consisting of narcosis, transition and reactivity. The selection principle of parameters for QSAR models was established and verified. It showed a reasonable prediction of toxicities caused by organophosphates and benzene derivatives, especially. Compared with traditional procedures, incorporating TRO showed an improved correlation coefficient of QSAR models by approximately 10%. Our study indicated that the proposed procedure can be used for screening modeling parameter data and improve the toxicity prediction by QSAR models, and this could facilitate prediction and evaluation of environmental contaminant toxicity.

## 1. Introduction

With the rapid development of the economy, more chemicals have been synthesized and used, and entered the ecosystem through environmental media such as atmosphere, water and soil, resulting in an augment of environmental pollutants and increase in ecological risk, and this leads to more work on the human health protection [1,2,3]. Toxicity data of environmental pollutants are mostly derived from animal experiments and model predictions. These data parameters include LC_50_ or LD_50_ (a concentration or dose to cause 50% death of exposed organisms), EC_50_ (a concentration to cause a 50% response) and NOEC (no observed effect concentration). Toxicity data for environmental pollutants are mostly derived from animal experiments and model predictions. However, with increasing concerns about animal welfare, in 1959, William Russel and Rex Burch proposed a 3R principle for animal experiments based on laboratory animal welfare [4]. The 3R principle referred to replacement, reduction and refinement, namely, replacement of animal use. In addition, toxicity data obtained from animal experiments can be unpredictable for human health risk due to species differences. In silico model prediction would become an alternative trend to assess toxic effects of environmental contaminants.

Quantitative Structure–Activity Relationship (QSAR) models have shown potential to completely predict toxic effects of contaminants and are used for risk assessment [5,6,7,8], for example, pharmaceuticals and personal care products (PPCPs), and aromatic compound and pesticides [9,10,11]. Model predictions can be used as an alternative to experimental tests (especially animal experiments) and also can provide useful information for the exploration of the mode of action (MOA) [12,13]. The current modeling approaches have demonstrated efficiency in handling collected datasets and these models include partial least squares regression (PLS), the Monte Carlo technique with two open source tools, namely small dataset modeler and CORAL software [14,15]. Ma et al. [16] used PLS to screen a QSAR model data set for 4-aminocinnoline-3-carboxamides, a Burton’s tyrosine kinase inhibitor, and the results showed that the prediction with this model was well performed. Amiri et al. [17] adopted a genetic algorithm (GA) for selected variable subsets and established a multiple linear regression model and artificial neural network model and achieved good prediction results. However, it was found that 80% of QSAR models (Table 1) exhibit a low correlation coefficient (R^2^ < 0.90), which would need further improvement. Pollutants with different active functional groups may show different properties in the biochemical reaction process, such as anilines and phenols. For example, the isolated electron orbital of aniline was conjugated with the benzene ring, and the electron cloud was dispersed on the benzene ring, thereby reducing the electron cloud density around nitrogen. According to density functional theory in the ground state of the molecular system, the electron density determined properties of the molecular structure and chemical reactivity [18]. A decrease in electron density may lead to a decrease in the chemical reaction activity of substituted aniline. Benzene was a representative compound of aromatic hydrocarbons, and its electrophilic substitution reaction was an example of its chemical properties [19]. Phenol may undergo a electrophilic substitution reaction similar to benzene on the ring due to the structure of the benzene ring. Increases in the electron cloud density of the benzene ring and chemical reaction activity account for the electron-donating effect of the hydroxyl group [20]. The prediction results of the QSAR model for phenolic contaminants were reasonable and were consistent with measured values (R^2^ > 0.90). Structural characteristic parameters of compounds can be affected by complex environments, which lead to deviations in toxicity end point prediction. Other contaminants, including volatile organic compounds, hydrocarbons, etc., exhibited low prediction due to various descriptors and complex influencing factors (R^2^ < 0.90).

The objectives of this study were to use a toxicity rank order (TRO) as a parameter to improve toxicology predictions of QSAR models and facilitate prediction and evaluation of toxicity caused by environmental contaminants. We collected toxicity data and established the principle of model parameter selection, compared the R^2^ of the model establishment before and after improvement, and then evaluated the accuracy and validity of the QSAR model. This study took a new approach and explored the feedback relationship between the prediction model and the classification of toxicity mechanisms. On the one hand, different modes of action corresponded to the discovery of large deviations from the model; on the other hand, the accuracy of the model was improved by determining the different action mechanisms of deviations.

## 2. Materials and Methods

### 2.1. Data Sources

The toxicity parameters cited in this paper were obtained from published literature on chemical toxicity [25,26,27,28]. Octanol-water partition coefficients (log K_OW_) of chemicals were estimated using an atom/fragment contribution method through the software of EPI suite.

### 2.2. Theory and Methodology

The QSAR model is a tool to predict toxicity endpoints through statistical analysis of the relationship between molecular structure and activity of artificial chemicals. The structure and properties of contaminants could mediate toxic mechanisms of contaminants to certain extent, and some related models of the structure and properties of substances can be determined. This was also verified in the toxicity prediction of fish and mice [29]. Due to the influence of different mechanisms on the prediction accuracy of the model, a good model should not only be based on structural properties, but also take into account the impact of environmental mechanisms. In order to further improve its accuracy, in the case of similar pollutant structure characteristics, different toxic modes can be obtained by using acute and chronic toxicity data through environmental mechanisms under different model organisms.

The QSAR model has been demonstrated to be an effective tool for analysis of toxic data of contaminants. TRO was a parameter based on quantitative toxicity endpoint values of LC_50_ and NOEC, and may serve as a better model and facilitate the prediction. The equation is shown in (1).

(1) TRO=LC50NOEC.

LC_50_ refers to the concentration that causes 50% lethality to organisms; NOEC refers to the maximum non-effect concentration of contaminants on organisms.

Most relevant modeling parameters were derived from experimental measurements and software calculations, and this calculation process was complex and the experimental reproducibility could be low, which caused uncertainty of model parameters and made parameters less valuable and the prediction more difficult. The TRO method was used by comparing acute and chronic toxicity data. A value of log TRO < 1 would indicate a MOA being narcosis and that toxicity was persistent with exposure time and accumulation amount. A value of log TRO > 1 would suggest a non-specific MOA, due to a reactive MOA in most cases, and toxicity might not be related to time or amount accumulation. Considering the systematic complexity, we proposed a transitional situation where a coexistence of the two kinds of toxicity mechanisms occur. Log TRO values of 1–3 would suggest a transition MOA. Toxicity data from different sources were affected by a variety of factors, such as biological species, testing methods, environmental effects, etc. If the MOA of organic contaminates were further accurately divided, unified correction and unification of toxicity data could be explored in future studies.

### 2.3. Approach Details

**Figure 1:**
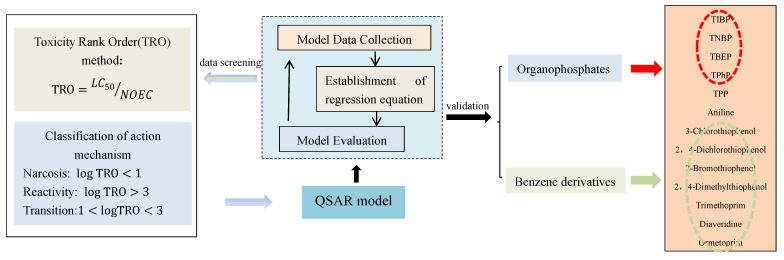
Flow chart of identifying abnormal toxicity endpoints and improving model effectiveness.

The approaches included data collection, data screening methods development, data screening modeling and model prediction. The following considerations were taken for development of the hypothesis, and the design framework is shown in Figure 1.

- Reviewed and collected the relevant toxicity literature for the QSAR model, identified the toxicity endpoints of model prediction, and used the TRO method to filter the modeling data.
- Summarized the modeling rules of QSAR and established the principles for parameter selection of QSAR models.
- Selected environmental contaminants according to QSAR model parameter selection principles for validation.

### 2.4. Validation Analysis

The toxicity endpoints and chemical descriptors for three contaminants were selected for validation analysis. The data were screened in advance based on the classification of toxicity mechanism types. The model was established according to the principle of modeling parameters for the QSAR model to verify the accuracy of TRO. The linear relationship between the QSAR model equation and pollutant prediction was determined using Microsoft Excel 2010. Accuracy improvement of the QSAR model was evaluated using correlation coefficients (R^2^). The percentage increase in R^2^ was equal to ($R_{\mathrm{im}}^{2}$ − $R_{\mathrm{in}}^{2}$)/$R_{\mathrm{in}\;}^{2}$ ×100% (${\;R}_{\mathrm{in}}^{2}$—correlation of initial data; $R_{\mathrm{im}}^{2}$—data correlation after improvement).

## 3. Results and Discussion

### 3.1. Definition and Calculation of Toxicity Rank Order

The predictions for contaminants by the QSAR model were generally based on the physicochemical properties and structural parameters of the contaminants such as molecular descriptors for quantitative and qualitative prediction. Molecular descriptors are mathematical symbols that characterize the molecular structure by the values of chemical compositions related to different physical or biological properties. The TRO definition refers to the use of measurable and predictable physical and chemical parameters of contaminants and toxic data to describe the level of toxic effects on organisms. It is a ratio of the acute and chronic toxicity data of contaminants to organisms: the LC_50_ for acute toxicity and NOEC for chronic toxicity. TRO was used in compound classification previously but did not exhibit good predictions [30].

The acquisition of TRO values was analyzed by taking organophosphate esters as an example, shown in Table 2. The TRO method provided benefits such as shortening the experimental cycle, saving funds, improving sensitivity, and reducing the consumption of a large number of experimental animals.

### 3.2. Classification of Toxicity Mechanism Based on TRO

The mode of action (MOA) has been recognized as a key determinant for pollutant toxicity [31,32]. However, MOA classification has never been standardized in ecosystems, and the comparison of different classification tools and methods has been rarely reported. In order to quickly identify the toxic categories of contaminants, the MOA of toxic contaminants was divided by the TRO method. There are many classification methods for the toxic mechanisms of contaminants, which are mainly divided into two categories, namely, narcosis and reactivity [33]. Narcosis-type contaminants are described as organic contaminants with the lowest toxicity to living organisms and are directly related to accumulation and of amount of accumulation [34]. The mechanism of narcosis likely results from a hydrophobic non-covalent interaction between contaminants and cell membranes, reversibly changing the structure and function of the cell membrane [35]. Reactivity-type contaminants have a mode of action that compounds themselves or their metabolites reacted with certain structures ubiquitously in biological macromolecules. The toxicity of transition-type contaminants exists between narcosis and reactivity. In the entire process of toxicity, it is affected by many aspects such as toxic effects, environmental behavior and biological properties (such as species, gender, age, individual, cell, target organ, etc.).

Narcosis was a mode of action (MOA), refers to a type of contaminant that may change relative to exposure concentration. It was found that toxicity for assessed contaminants was highly correlated with log K_OW_ [36,37]. When log TRO ≥ 3, the pollutant MOA was a reactive type of pollutant. Reactive MOA refers to a type of contaminant that is toxic to organisms and associated indirectly with exposure time and accumulation amount. This is typically a specific MOA which may be highly correlated with quantum parameters presenting reactive activity. There was, moreover, a need to recognize the variation and complexity in chemical toxic modes of action; the same compound may act via different modes in different organisms, at different life-stages, at different concentrations, and at different targets. Thus, there would be a mid-state of MOA with co-existing narcosis and reactive action referred to as transition. The TRO method was proposed to identify the possible MOAs by finding the deviation values from QSAR prediction. We proposed a transitional situation, in which the present cut-off value of log TRO was 1–3. For contaminants with transitional toxic effects, the correction factor method was used to correct them in the later stage, so it could be redefined by the TRO method, for the prediction and evaluation of contaminants in the QSAR model. In terms of toxicity mechanism, most studies have defined the toxicity level of compounds to organisms as the accumulation of quantities. There was moreover a need to recognize the variation and complexity in chemical toxic modes of action, the same compound may act via different modes in different organisms, at different life-stages, at different concentrations, and at different targets.

### 3.3. Rule of Parameter Selection

The development of QSAR models with appropriate modeling parameters helps to avoid the over-fitting and poor fitting performance of QSAR models. Therefore, selection of modeling parameters is pivotal for the establishment of QSAR models and directly impacts the quality of models. Based on the TRO method, the toxicity endpoints were screened. Firstly, according to TRO by definition, reactive compounds were found to exhibit strong biological toxicity, complex environmental behavior and poor biodegradability. It was difficult to use a single toxicity data set to model and predict the toxicity mechanism of a two-dimensional QSAR model. We selected common quantum chemical descriptors to model reactive type of contaminants. Molecular descriptors were also used to analyze toxic pesticides on *Skeletonema costatum* [38]. The results showed that the regression and classification models were robust with reasonable prediction, and relatively high sensitivity. The toxicity mechanism modes of contaminants were associated not only with K_OW_, but also with the quantum parameters and three-dimensional space structure for target compounds. The K_OW_ determines the bio-uptake potential of a hydrophobic compound and closely correlated with toxicity levels. On the other hand, the quantum parameters determine the reactivity and the specific action between contaminants and biological receptors. In this study, K_OW_ was used as a descriptor with which to construct two-dimensional QSAR models for narcosis-type contaminants. Three dimensional QSAR models broadly represent all such properties of atoms in reactive contaminants that are represented by quantum chemical descriptors. Narcosis-type contaminants exhibiting weak biological toxicity and strong biodegradability were closely associated with K_OW_. Since environmental behavior was relatively simple and predictable, and acute and chronic toxicity data were often used for modeling, simple two-dimensional descriptors were selected for the model and analyzed for acute oral toxicity of polycyclic aromatic hydrocarbons (PAHs) in rats [39]. It was found that a QSAR model using LD_50_ could be established. Two-dimensional and three-dimensional QSAR models are shown in Table 3.

### 3.4. Validation of Application in Selected Case

**Figure 2:**
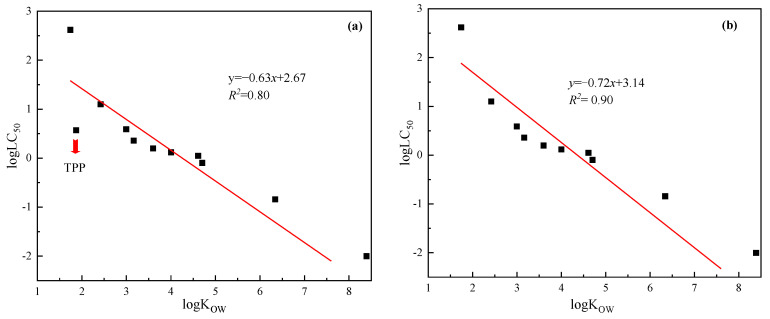
Model fitting curve of QSAR model for organophosphates ((**a**)—the fitting effect of QSAR model without TRO method; (**b**)—the fitting effect of QSAR model with TRO method; The red line showed the fitting effect of the model).

The organic phosphates were selected for construction of linear regression equations with their log K_OW_ and log LC_50_ as X-axis and Y-axis, respectively. Figure 2a,b represents the fitting of the QSAR model with and without the TRO method, respectively.

The effectiveness of the established QSAR model for organophosphate esters was analyzed. As shown in Figure 2, the prediction fitting degree of the QSAR model was significantly improved using the TRO method, as evidenced by a higher R^2^ of 0.90 (Figure 2b) and lower standard deviation of errors of prediction, as compared with the model without the TRO method (Figure 2a, R^2^ = 0.80). The results showed that the TRO method was feasible and reliable for the identification of model prediction deviation values and for improvement of model validity. In addition, biological toxicity was effectively distinguished by TRO classification. For example, organophosphate ester contaminants (Table 2), exhibited mostly narcosis; biological toxicity was evident when they accumulated to a certain concentration. Studies have shown that tributyl phosphate (TBP), TPP and tris (2-butoxyethyl) phosphate (TBOEP) exhibit similar concentration dependence at the cellular level, while TBP and TBEP are more easily degraded than TPP, leading to different properties and greater toxicity [45]. For anesthetic pollutants, the toxicity was lower, but due to accumulation capability, they can cause a greater impact on organisms.

**Figure 3:**
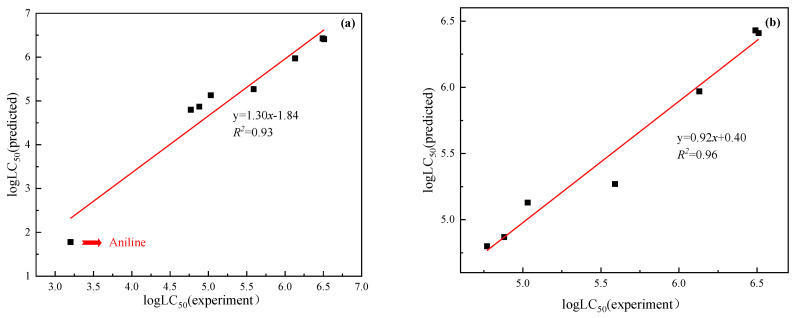
QSAR-model fitting correlation curve between predicted and experimental values of benzene derivatives ((**a**)—Correlation curve between predicted LC_50_ and experimental LC_50_ without TRO method; (**b**)—Correlation curve between predicted LC_50_ and experimental LC_50_ by TRO method; The red line showed the fitting effect of the model).

The selected modeling parameters of benzene derivatives are shown in Figure 3. The scatter plots were developed to compare the correlation between biological activity and its corresponding octanol water partition coefficient.

The TRO method was used to screen contaminant benzene derivatives. The results showed that the degree of fitting for the predicted and experimental values of LC_50_ of benzene derivatives using the QSAR model with the TRO method significantly increased by 4%, indicating an effective improvement in the efficiency and accuracy of QSAR modeling, and this was significant for the evaluation and prediction of pollutant toxicity effects. The regression coefficient (R^2^) of 0.96 between observed and predicted LC_50_ parameters showed good prediction by the developed QSAR model. The selected list of benzene-derivative toxicity-endpoint values along with classification result are listed in Table S1.

### 3.5. Comparison of Model Performance with TRO

In order to evaluate the effectiveness of the TRO method for the modeling of QSAR models, the correlation coefficients of model prediction for the common QSAR models (Table 1) were evaluated using an external test (Table 4). According to the model R^2^ value, the model R^2^ that was not promoted by the TRO method was taken as the control group and the model R^2^ that was promoted by the TRO method was taken as the correction group. The relative standard deviation before and after correction represented the improvement effect of R^2^. The results showed an improvement by a maximum of 10%, and the correlation coefficients were increased to more than 0.90. The validity of the TRO method, the rationality of the toxicity mechanism classification and the accuracy of model prediction were verified as shown in Table 4.

As shown in Table 4 above, significant improvement was observed for model predictions for all different kinds of contaminants by using the TRO method of toxicity data screening. Among them, the QSAR model of volatile organic molecules with the lowest prediction performance was increased by 8.64%, from 0.74 to 0.81. Artificial products with good prediction results were also obtained, to a certain extent. This showed that the TRO method can be successfully applied to the establishment of QSAR model data screening and improve the effectiveness of model prediction.

## 4. Conclusions

In this study, a new approach using TRO modeling parameters has been proposed that allows successful prediction of QSAR models. The model showed good prediction, especially in organophosphates and benzene derivatives. Based on the TRO method, the boundary values of the three toxicity modes of contaminants were distinguished, and the toxicity mechanisms were classified into reactivity, transition and narcosis. The usefulness of the TRO approach for other emerging contaminants such as nanoparticles and micro-plastics will be examined in further studies. Further development of reasoned methods is needed to elucidate and differentiate the definition domains for MOA in complicated systems with different samples and model organisms. The application of the TRO method to the assessment of the MOA of organic contaminates is beneficial to the evaluation of ecological risk and helps to explain the toxicity mechanism of organic contaminates.

## Figures and Tables

**Table 1:** QSAR models for common toxicity prediction.

No.	Compound	Equation	R^2^	Ref.
1	Volatile organic molecules	pNOAEC*=6.906-0.605×nF-0.044×H%+1.802×Psii0d+2.065×X3A-0.2897×nR	0.74	[5]
2	Phenols	TEAC ** = ∑(αi – αi *) × exp[−0.6 × (|X − Xi|) 2] + 0.758	0.90	[21]
3	Phthalate acid esters	log K=13.82+0.961EHOMO+2.529Qc − 0.503logSOM+0.064pH	0.89	[22]
4	Natural Products	${\log(\mathrm{IC}}_{50})\;***=115.86\;-\;0.24\mathrm{FHFA}+5.4\mathrm{MACMO}$	0.91	[23]
5	Organic compounds	${\log\;\mathrm{LC}}_{50}=$ −4.08+0.065 minsOH+0.123 minHBintt2+0.834GATS5s+0.196MAXDP −0.029VE3_DzZ+57.17VE2_Dt+1.728GATS1p+0.13SaasC − 0.055C3SP2	0.82	[24]

* No observed adverse effect concentration (NOAEC); ** Trolox-equivalent antioxidant capacity (TEAC); *** 50% concentration of inhibition (IC_50_). Other model parameters were shown in Table S2.

**Table 2:** Information on related parameters of organophosphates.

Compound	CAS	Abbreviation	log K_OW_	log LC_50_ (mg·L^−1^)	log NOEC (mg·L^−1^)	log TRO	Types
Triisobutyl phosphate	126-71-6	TIBP	3.60	0.20	0.50	0.30	N *
Tributyl phosphate	126-73-8	TNBP	4.00	0.12	0.24	0.12	N
Tributoxyethyl phosphate	78-51-3	TBEP	3.00	0.59	1.40	0.81	N
Triphenyl phosphate	115-86-6	TPHP	4.70	−0.10	−0.73	0.63	N
Diphenyl chlorophosphate	2524-64-3	-	3.16	0.36	1.03	0.67	N
Tripropyl phosphate	513-08-6	TPP	1.87	0.57	1.90	1.36	T **
Diethyl phthalate	84-66-2	DEP	2.42	1.10	0.79	0.30	N
Di-n-butyl phthalate	84-74-2	DNBP	4.61	0.05	−0.25	0.30	N
Bis(2-ethylhexyl)phthalate	117-81-7	BEHP	8.39	−2.0	−2.30	0.30	N
Bis(2-butoxyethyl) phosphate	14260-97-0	BBOEP	1.74	2.62	2.32	0.30	N
Tricresyl phosphate	1330-78-5	TCP	6.34	−0.84	−8.15	0.30	N
Diethyl phthalate	84-66-2	DEP	2.42	1.10	0.79	0.30	N

* N: narcosis; ** T: transition.

**Table 3:** Common applications of QSAR with different dimensions in environmental contaminants.

No.	Type	Compound	Equation	R^2^	Ref.
1	2D- QSAR	Dioxin	${\mathrm{pEC}}_{50}=8.827+2.047E1m+2.156\mathrm{SM}09\_\mathrm{AEA}(\mathrm{dm})-0.306$ RDF065u−0.598F05[Cl-Cl] −0.817Neoplastic-80	0.91	[40]
2		MKC-442	${\mathrm{pEC}}_{50}=8.24130.40315\mathrm{CHIV}3P+0.1618\mathrm{DipoleX}+3.131$ 4Jurs_FNSA2+0.90452Kappa3AM−0.024 976ShadowYZ	0.79	[41]
3	3D- QSAR	β2-adrenocep	${\mathrm{pEC}}_{50(\mathrm{pre})}=0{.9907\mathrm{pEC}}_{50(\exp)}+0.2017$	0.99	[42]
4		Phenylpyrrole Fungicides	${\mathrm{pEC}}_{50(\mathrm{pre})}=0{.98731\mathrm{pEC}}_{50(\exp)}+0.050207$	0.99	[43]
5		Phthalate Esters	$h_{i}=x_{i}\left(\mathrm{XTX}\right)-{\;X}_{i}T$	0.99	[44]

**Table 4:** Improved QSAR models for common toxicity prediction based on Table 1.

**No.**	**Compound**	Equation	$R_{\mathrm{in}}^{2}*$	$R_{\mathrm{im}}^{2}**$
1	Volatile Organic Molecules	pNOAEC=6.906−0.605 × nF−0.044×H%+1.802×Psii0d+2.065 × X3A −0.2897×nR	0.74	0.81
2	Phenols	TEAC=∑(αi – αi*)×exp[−0.6 × (|X − Xi|) 2] + 0.758	0.90	0.91
3	Phthalate Acid Esters	log K=13.82+0.961EHOMO+2.529Qc−0.503logSOM+0.064pH	0.89	0.92
4	Natural Products	${\log(\mathrm{IC}}_{50})=115.86-0.24\mathrm{FHFA}+5.4\mathrm{MACMO}$	0.91	0.91
5	Freshwater Crustacean	${\mathrm{logLC}}_{50}=-4.08+0.065\mathrm{minsOH}+0.123\mathrm{minHBint}2+0.834\mathrm{GATS}5s+0.196$ MAXDP−0.029VE3_DzZ+57.17VE2_Dt+1.728GATS1p+0.13SaasC− 0.055C3SP2	0.82	0.87

* $R_{\mathrm{in}}^{2}$—correlation of initial data; ** $R_{\mathrm{im}}^{2}$—data correlation after improvement.

## Data Availability

The data presented in this article are available on request from the corresponding authors.

## Supplementary Materials

The following supporting information can be downloaded at: https://www.mdpi.com/article/10.3390/ijerph20010701/s1, Table S1: Acute and Chronic Toxicity Data of Benzene Derivatives. Table S2: Summary of the descriptors appearing in the article.
