# Supplementary material for: Toxicity Rank Order (TRO) As a New Approach for Toxicity Prediction by QSAR Models
Source: Int J Environ Res Public Health. 2022 Dec 30;20(1):701. doi: 10.3390/ijerph20010701 (PMC9819504; doi:10.3390/ijerph20010701)
Supplement: Supplementary file 1 [file ijerph-20-00701-s001.zip › ijerph-2054934-supplementary.pdf]

Table S1 Acute and Chronic Toxicity Data of Benzene Derivatives

Compounds	CAS	-log EC_{50}/ mol/L		-log NOEC /mol·L-1	log TRO	Types
		Experimental value	Calculated value			
3-Chlorothiophenol	2037-31-2	5.03	5.13	1.70	3.33	R*
2，4-Dichlorothiophenol	1122-41-4	5.59	5.27	1.70	3.89	R
Aniline	62-53-3	3.20	1.78	1.70	1.54	T*
2-Bromothiophenol	6320-02-1	4.88	4.87	1.70	3.18	R
2，4-Dimethylthiophenol	13616-82-5	4.77	4.80	1.70	3.07	R
Trimethoprim	738-70-5	6.49	6.43	1.71	4.78	R
Diaveridine	5355-16-8	6.13	5.97	1.71	4.42	R
Ormetoprim	6981-18-6	6.51	6.41	1.71	4.80	R

*T: transition; R: reactivity

Table S2. Summary of the descriptors appearing in the article.

Descriptors	Explanation
nF	a constitutional index, describing the total number of fluorine (F) atoms present in a molecule
H%	a constitutional index, describing the percentage of H atoms in a molecule
Psii0d	a constitutional index, describing the total number of fluorine (F) atoms present in a molecule
X3A	X3A is average connectivity index of order 3
nR	a “functional group count” type descriptor
B01[C-O]	a 2D atom pair descriptor. It indicates whether a C (carbon) and an O (oxygen atom) are present at a topological distance 1 or not
B02[C-N]	a 2D atom pair descriptor, which indicates whether a carbon and a nitrogen atom are separated in a molecule by a topological distance 2 or not
B04[C-O]	a 2D atom pair descriptor, indicating whether a carbon and an oxygen atom are separated in a molecule by a topological distance 4 or not
B05[C-N]	a 2D atom pair descriptor, indicating whether a carbon and a nitrogen atom exist at the topological distance 5 or not
LogK	the degradation rate
E_{HOMO}	occupied molecular orbital energy
log SOM	soil organic matter
$\alpha i - \alpha i^*$	the Lagrange coefficient corresponding to the 24 support vectors
GATS1p	Geary autocorrelation of lag 1
GATS5s	Geary autocorrelation of lag 5
MAXDP	the maximum electrotopological positive variation
SaasC	an electrotopological descriptor that accounts for the number of carbon atoms that make two aromatic bonds and one single bond.
C3SP2	unded carbons linked to three other carbons and flags for the presence of poly-aromatic or unsaturated branched

	aliphatic systems.
minHBint2	the minimum E-State descriptor of strength for potential Hydrogen Bonds (HB) of path length 2
ATSC7v	the average centered Broto-Moreau autocorrelation of lag 7 weighted by van der Waals volumes
VE3_DzZ	weighted on the atomic number
FHFA	Final heat of formation
MACMO	Max antibonding contribution of a MO
E1m	WHIM descriptors
SM09_AEA(dm)	Edge adjacency indices
RDF065u	RDF descriptors
Neoplastic	Drug-like indices
F05[Cl-Cl]	2D atom pairs
